# Iron in Porphyrias: Friend or Foe?

**DOI:** 10.3390/diagnostics12020272

**Published:** 2022-01-21

**Authors:** Elena Buzzetti, Paolo Ventura, Elena Corradini

**Affiliations:** 1 Internal Medicine and Centre for Hemochromatosis and Heredometabolic Liver Diseases, ERN-EuroBloodNet Center for Iron Disorders, Azienda Ospedaliero-Universitaria di Modena-Policlinico, 41124 Modena, Italy; paolo.ventura@unimore.it; 2 Department of Medical and Surgical Science for Children and Adults, University of Modena and Reggio Emilia, 41124 Modena, Italy

**Keywords:** iron, heme, porphyrias, hepcidin, anemia

## Abstract

Iron is a trace element that is important for many vital processes, including oxygen transport, oxidative metabolism, cellular proliferation, and catalytic reactions. Iron supports these functions mainly as part of the heme molecule. Heme synthesis is an eight-step process which, when defective at the level of one of the eight enzymes involved, can cause the development of a group of diseases, either inherited or acquired, called porphyrias. Despite the strict link between iron and heme, the role of iron in the different types of porphyrias, particularly as a risk factor for disease development/progression or as a potential therapeutic target or molecule, is still being debated, since contrasting results have emerged from clinical observations, in vitro studies and animal models. In this review we aim to deepen such aspects by drawing attention to the current evidence on the role of iron in porphyrias and its potential implication. Testing for iron status and its metabolic pathways through blood tests, imaging techniques or genetic studies on patients affected by porphyrias can provide additional diagnostic and prognostic value to the clinical care, leading to a more tailored and effective management.

## 1. Introduction

Iron is a microelement of pivotal importance for both cells and organisms; it is essential for many vital processes such as energy production, biosynthesis, replication, and immune system functions.

Iron is necessary for the synthesis of heme, a cyclic tetrapyrrole prosthetic group that is deputized to link oxygen, which is contained in several essential proteins, the principal being hemoglobin (Hb), followed by myoglobin (Mb), catalases and cytochromes; such proteins are involved in oxygen transportation through the bloodstream to the tissues, oxygen storage in muscle cells, hydrogen peroxide degradation, and respiratory and energetic processes in the mitochondria, respectively.

Heme production mainly takes place in erythroid precursors and hepatocytes, reflecting the greater demands of heme in the bone marrow and the liver.

Porphyrias consist of a group of rare, either inherited or acquired, metabolic disorders that are ascribable to an alteration in one of the eight steps in the process that converts simple, linear amino-acid-like compounds (glycine/succinyl-Coenzyme A) to heme. To date, eight different types of porphyrias have been described, each caused by the defective activity of one of the eight enzymes involved in the heme biosynthetic pathway [1].

The common pathological denominator in all porphyrias is the consequent accumulation, and therefore increased elimination via the urinary or gastrointestinal tract, of porphyrins or their precursors, with varying clinical manifestations (including neurovisceral symptoms and skin lesions) depending upon which enzyme is affected.

Despite the strict biological link between iron and heme, the role of iron as a risk factor for disease development or progression, a potential treatment target, or, conversely as a potential therapeutic in porphyrias, has long been debated with contrasting results.

This review aims to deepen this aspect by focusing on the current knowledge on iron metabolism and its potential implication in the pathophysiology and clinical management of porphyrias.

## 2. Overview on Iron Metabolism

Although iron is necessary for several cellular functions, its excess is toxic. Due to its unique ability to switch between ferric and ferrous forms and vice versa via the Fenton and Haber–Weiss reactions, respectively, iron is both an ideal redox-active cofactor for important physiologic activities and a potential trigger for oxidative damage. When not bound by specific carriers or storage proteins, free iron is able to generate, through oxygen reduction, highly reactive intermediates called reactive oxygen species (ROS). Normally, low amounts of ROS are involved in physiological processes and biological responses [2]. However, in the condition of iron overload, when the antioxidant capacity of the cell is overcome, excessive ROS can damage proteins, nucleic acids and carbohydrates and cause the propagation of lipid peroxidation and cell apoptosis, leading to the initiation or progression of fibrogenesis and carcinogenesis, and to cell death. Iron-overload-related immunologic aberrancies have also been described, including altered macrophage immunosurveillance and immunomodulatory functions [3]. 

Since there are no actively regulated mechanisms for iron secretion when it is in excess, a strict balance between its uptake, transport, storage, and use is important in order to preserve iron homeostasis and prevent iron-related toxicity.

In the human body, circulating iron derives from three primary sources: recycling from heme of senescent erythrocytes, intestinal adsorption from the diet and release from the liver, which represents its main storage site. Under physiological conditions, the contribution of dietary iron mostly serves to compensate for iron losses. Iron recycling takes place in erythro-phagolysosomes of macrophages, which are localized mostly in the bone marrow, spleen, and the liver; after Hb catabolism, heme is exported into the cytosol, where it is catabolized by a microsomal enzyme, heme oxygenase (HO), into bivalent ferrous ions (Fe^2+^), carbon oxide (CO), and biliverdin IXa, which is subsequently reduced to bilirubin by biliverdin reductase [4]. To date, two isoforms of HO, encoded by two separate genes, have been described in humans: HO-1, whose activity can be induced by a series of factors (cellular stress, reactive oxygen species, heme, thermal shock, UV radiation, nitric oxide (NO), pro-inflammatory cytokines and heavy metals) in order to protect cells from the toxic and oxidant activity of free heme; and HO-2, which is constitutively expressed. The critical role of HO-1 in human physiology has been recently highlighted by case reports of children with genetic disruptions of its function and fatal outcomes [5].

Inorganic dietary iron is adsorbed in the duodenum and proximal jejunum by divalent metal cation transporter 1 (DMT1) after reduction to ferrous iron by a ferric reductase (duodenal cytochrome B, Dcytb) at the apical membrane of enterocytes [6]. Furthermore, the possibility of dietary ferritin absorption has also been suggested [7]. 

Iron is then exported from macrophages and enterocytes into the bloodstream via the iron exporter ferroportin (Fpn) [8] after it is transformed from the ferrous into the ferric form by ferroxidases, such hephaestin (Heph), which co-localizes with Fpn in enterocytes, or ceruloplasmin (Cp) in other cells types (e.g., macrophages, hepatocytes and astrocytes), in order to be bound to serum transferrin (Tf). 

Once iron is bound to Tf, its uptake from cells involves Tf binding to its specific receptor (TfR1) on the target cell’s surface, triggering an endocytic internalization culminating in the release of iron into the cytosol via DMT1. On the other hand, when excess iron exceeds the buffering capacity of Tf, free iron can easily enter the cells via TfR1-independent pathways. Indeed, transporters for non-transferrin-bound iron (NTBI) such as calcium-channels, DMT1, solute carrier family 39 member 8 and member 14 (ZIP 8 and ZIP 14) have been identified in cell cultures and murine models [9] but their relevance in humans has not been well characterized yet. 

NTBI represents an important pathological marker of iron overload and potential toxicity, particularly in diseases such as thalassemia or other types of transfusion-dependent anemias, although the type of NTBI and its level relevant to the pathological loading of tissue compartments still have to be defined [10].

Once inside the cell, iron can be stored in the cytosol and assembled with ferritin (Ft) or used within the mitochondria. In vertebrates, cytoplasmic Ft is expressed in almost all tissues, and it consists of 24 subunits of heavy (H) and light (L) chains in various ratios and can sequester 4.500 iron atoms [11]. 

The mechanisms by which iron is acquired by mitochondria are not completely understood. Several mechanisms seem to contribute, comprising the direct delivery of extracellular iron to the mitochondria (in a sort of ‘kiss and run’ mechanism) and the mitochondrial uptake of cytosolic iron [12]. Proteins acting as chaperones may also be involved in intracellular iron trafficking [13]. The transport of iron across the internal mitochondrial membrane is an active process and the principal iron importers at this site, in a variety of tissues, are mitoferrin-1 (Mfrn), which is known to interact with ATP binding cassette subfamily B member 10 (ABCB10), an ATP-binding-cassette protein which enhances Mfrn stability and mitochondrial iron import in erythroid cells [14], and its homolog Mitoferrin-2 [10], although the exact mechanism remains obscure. In the mitochondria, iron is mainly utilized for two metabolic pathways, heme synthesis and iron–sulfur-cluster biogenesis, but can also undergo mitochondrial storage. Mitochondria are also prone to iron-driven damage when its intracellular or intra-organelle level becomes toxic [15].

**Figure 1:**
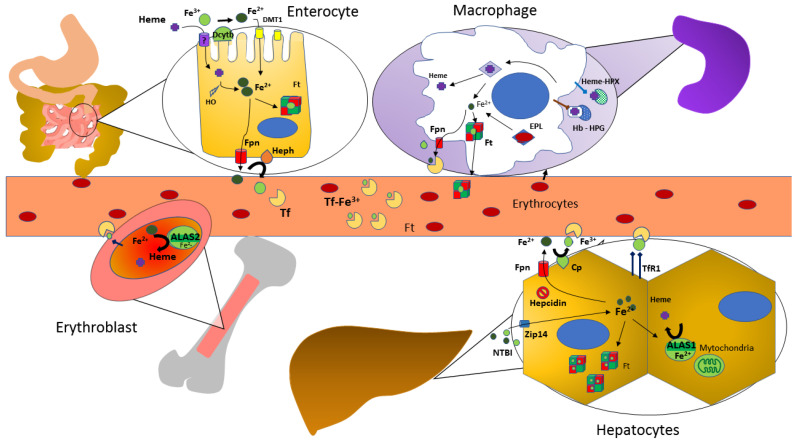
Iron Metabolism in the Human Body. Abbreviations: DMT-1, divalent metal transporter 1; Dcytb, duodenal cytochrome B; HO: heme oxygenase; Ft, ferritin; Fpn, ferroportin; Heph, hephaestin; Tf, transferrin; EPL, erythrophagolysomes; Cp, ceruloplasmin, ferroxidase activity; Hb, hemoglobin; HPX, hemopexin; HPG, haptoglobin; NTBI, non transferrin-bound iron; TfR1, transferrin receptor 1; ALAS, aminolevulinate synthase.

A visual summary of iron metabolism in the human body is depicted in Figure 1.

Iron excretion is an unregulated process that occurs through enterocyte turnover, the shedding of hair and skin cells, loss in sweat, menstruation in women and minor bleeding. Although more recently it has been hypothesized that iron could also potentially enter the human body via the respiratory tract (as observed in subjects with prolonged professional exposure to welding fume) [16], its adsorption in the intestinal tract is the only step of iron exchange with the environment which is actively controlled by body’s iron status, and this is mainly achieved by ferroportin regulation.

Therefore, circulating iron levels depend on iron intestinal absorption and iron release from the reticuloendothelial system, with Fpn functioning as the limiting step of both.

Ferroportin regulation is quite complex, and can occur at the transcriptional, post-transcriptional, or post-translational level [17]; hepcidin, the so-called ‘iron hormone’, is its main negative regulator and is therefore central to iron homeostasis.

Hepcidin (coded by the HAMP gene) is a small peptide, mainly synthesized in the hepatocytes; it is released into the circulation and binds to Fpn at the surface of several cells, including enterocytes, macrophages and hepatocytes, leading to its ubiquitination, internalization and lysosomal degradation [18]. More recently, it has been suggested that hepcidin can also occlude the central cavity of Fpn and thereby interfere with iron export independently of endocytosis. This mechanism could be particularly relevant in cells expressing high levels of Fpn but lacking endocytic machinery such as red blood cells [19].

The constitutive expression of hepcidin in the liver relies on an intact bone morphogenetic proteins (BMP)—mothers against decapentaplegic homologs (SMAD) (pathway, with a pivotal role of proteins located on the cell membrane such as hemojuvelin (HJV), homeostatic iron regulator (HFE) and transferrin receptor 2 (TfR2), which act as a co-receptor and auxiliary factors, respectively [20]. 

In cases of increased tissue iron, the expression of bone morphogenetic proteins 6 and 2 (BMP 6, BMP 2) increases in hepatic sinusoidal endothelial cells, with the consequent activation of the HAMP gene promoter in the hepatocytes and enhanced protein production and release into the bloodstream; as a consequence, hepcidin binds to Fpn, leading to its internalization and degradation.

It seems that hepatic-tissue iron content at least partially regulates hepcidin production, thereby regulating the BMP6 and BMP2 ligands syntheses, whereas serum iron induces hepcidin by activating hepatocyte 1,5,8 mothers against decapentaplegic homologs 1, 5, 8 (SMAD 1, 5, 8) signaling downstream or independent of an increase in BMP2/6 ligand [21]. Recently, nuclear factor erythroid-derived 2-like 2 (NRF2) has been linked to the above mechanism. It has, in fact, been shown that this transcription factor is activated by iron-induced, mitochondria-derived pro-oxidants, and it enhances BMP6 expression in hepatic sinusoid endothelial cells with consequently increased hepcidin production, suggesting that NRF2 could link the cellular sensing of excess toxic iron to the control of antioxidant responses and systemic iron homeostasis [22].

Overall, this molecular machinery is aimed at protecting the body from both circulatory- and tissue-iron overload and subsequent iron-driven cell damage. 

Matriptase-2 is a serine protease encoded by the TMPRSS6 gene, which exerts its negative activity on hepcidin by cleaving from the membrane the GPI-anchored co-receptor HJV, thereby suppressing BMP-SMAD signaling and hepcidin transcription [23]. It has been shown that iron deficiency not only prevents matriptase-2 degradation, leading to hepcidin suppression, but also increases the membrane HJV cleavage by the furin family of proprotein convertases, thereby generating a form of soluble HJV that is able to bind and sequester BMP ligands [24].

Although it was originally described that TMPRSS6 is increased/stabilized by iron deficiency, erythropoietic drive or hypoxia, it has been shown that chronic iron overload and BMP6 levels can also induce TMPRSS6 expression, and such modulation could lead to a negative feedback mechanism to avoid an excessive stimulation of hepcidin induced by iron in order to maintain iron homeostasis [25].

Hepcidin is also positively regulated by inflammation, particularly by interleukin-6 (IL-6) via the signal transducer and activator of transcription 3 (STAT3)- janus kinase 1/2 (JAK1/2) pathway as part of the innate protective mechanisms that are activated in response to infections, in order to induce iron sequestration in the body stores and to prevent its utilization by micro-organisms [26]. Other modulatory signals for hepcidin have been described, such as growth factors and sex hormones [27].

If hepcidin’s synthetic machinery fails to respond to such regulatory pathways or in the case of pathological levels or activity of such signals or pathways, a disturbance in iron homeostasis can occur, leading to the development of iron overload, iron deficiency or iron misdistribution. In hereditary hemochromatosis, mutations in genes such as HFE, TFR2, HJV, HAMP cause a hepcidin deficiency due to the reduced activity of the hepatic BMP-SMAD-signaling pathway or hepcidin synthesis per se, resulting in increased iron adsorption and iron release into the bloodstream [5]. Hepcidin level is also reduced in iron-loading anemias, wherein erythroferrone (ERFE) that is released from the bone marrow of subjects with ineffective erythropoiesis has been identified as the main suppressor of HAMP expression, likely via the SMAD-signaling pathway, although the mechanisms need to be fully understood [28].

Vice versa, an increased expression of HAMP leads to circulating-iron restriction; this is a major pathophysiological mechanism underlying the anemia of inflammation, wherein pro-inflammatory interleukin-6 raises hepcidin levels through the STAT pathway, or in iron-refractory iron-deficiency anemia (IRIDA), wherein mutations of the hepcidin inhibitor TMPRSS6 up-regulate the BMP-SMAD pathway [25].

Another key mechanism in iron homeostasis, acting at the cellular level, is the iron-regulatory-protein/iron-response-element (IRP/IRE) machinery. Iron regulatory proteins 1 and 2 (IRP1 and IRP2) modulate several iron proteins at the post-transcriptional level through a mechanism that involves their binding to sequences in the untranslated regions of mRNAs (IREs), consequently controlling mRNA translation and stability. For instance, under the conditions of iron starvation, IRPs stabilize the mRNAs encoding for TFR1 and inhibit the translation of ferritin and ferroportin mRNAs; they also control the expression of other proteins involved in iron and energy metabolism [29]. Splicing variants of ferroportin mRNA lacking the 5′-IRE, which are therefore not responsive to intracellular iron depletion, have been described in duodenal enterocytes and erythrocyte progenitor cells, highlighting the multistep and multilevel control of iron homeostasis [30].

Hypoxia inducible factors (HIFs) are also involved in intracellular iron homeostasis regulation. HIF-1 is the most extensively studied subunit so far, and it has been shown to regulate TfR1 and HO-1 expression [31,32]. HIF-2 has a relevant role in erythropoiesis by regulating the erythropoietin hormone (EPO); it also increases iron mobilization by different mechanisms: in the liver specific HIF-2 activation indirectly inhibits hepcidin synthesis through the EPO-mediated stimulation of erythropoiesis, while in the intestine, HIF-2 activates the transcription of DMT1, CYBRD1 (coding for DcytB), and FPN [33]. HIF-2 itself is also an IRP target [34].

A recently described mechanism that participates in intracellular iron homeostasis is represented by nuclear-receptor-coactivator-4 (NCOA4)-mediated ferritinophagy; this cargo receptor or chaperone facilitates ferritin degradation or iron storage according to cellular demands and it also seems to be involved in ferroptosis, an iron induced cellular death that can occur in situations of increased or unregulated ferritinophagy and therefore increased lipid peroxidation by labile cellular iron [35]. 

## 3. Heme Metabolism, Iron and Reciprocal Influences

Heme production within the body mainly takes place in erythroid precursors in the bone marrow and in hepatocytes. An accurate description of the process and its regulation is better reported elsewhere in other reviews [36].

Briefly, the first step is the production of ALA, which is catalyzed by ALA synthase (ALAS) in the mitochondrial matrix. Of the two isoforms of ALAS, the isoform 1 (ALAS1) is expressed ubiquitously and is involved in heme synthesis in cells other than erythroid precursors, while the isoform 2 (ALAS2) is encoded by a gene located on the X chromosome and is expressed only in erythroid cells, particularly in the late stages of red-blood-cell maturation. Its induction is fundamental and finely regulated.

Once formed, ALA is exported into the cytosol and converted by ALA dehydratase (ALA-D) to porphobilinogen (PBG), which is in turn converted to hydroxymethylbilane (HMB) by PBG deaminase (PBG-D), then to uroporphyrinogen III (UroPIII) by uroporphyrinogen III synthase (UROS); UroPIII becomes coproporphyrinogen III (CPPIII) through the activity of uroporphyrinogen III decarboxylase (UROD). CPPIII is then imported into the mitochondrial intermembrane space where it is converted to protoporphyrinogen IX (ProtoIX) by coproporphyrinogen oxidase (CPOX); protoporphyrinogen IX is oxidized to protoporphyrin IX (PPIX) by protoporphyrinogen oxidase (PPOX). At this step, the enzyme ferrochelatase (FECH) incorporates iron into PPIX to form heme. 

On the contrary, the process of dietary-heme uptake has not yet been well characterized. A receptor-mediated endocytosis in the gut has been proposed, as it is more likely than passive diffusion and it seems a more efficient process than inorganic-iron adsorption [37]. Dietary heme, at the acidic pH present in the stomach, is dissociated from hemoproteins [38] and is absorbed mostly in the proximal intestine. The mediation of heme carrier protein 1 (HCP1) and hephaestin has been previously proposed, with contrasting results, and still remains obscure [39,40].

**Figure 2:**
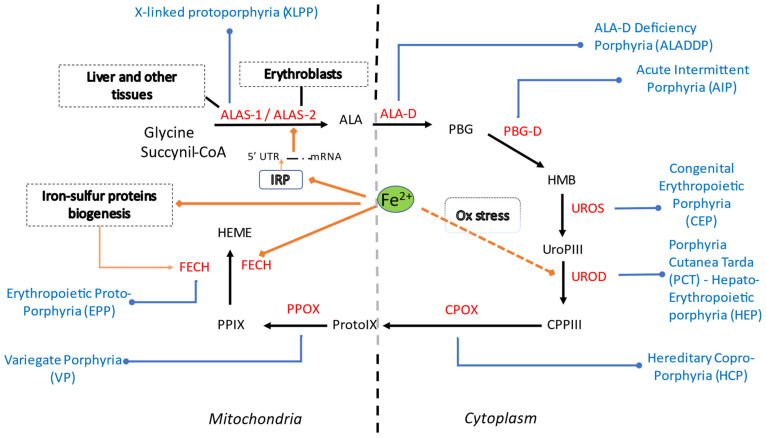
Summary of heme synthetic pathway, types of porphyrias and iron influences within the cell (cytosolic and mitochondrial compartments). Heme synthesis starts in mitochondria, with the condensation of succinyl-CoA with the amino acid glycine, activated by pyridoxal phosphate by ALA synthase, the rate-limiting enzyme of heme synthesis. ALA then enters the cytoplasm, where, in the presence of ALA dehydratase, porphobilinogen and water molecules are formed. Four PBG molecules are joined by PBG deaminase as hydroxymethylbilane. Linear tetrapyrrole cyclizes to form a ring known as uroporphyrinogen III with the participation of uroporphyrinogen III synthase. Through the activity of uroporphyrinogen III decarboxylase (UROD), coproporphyrinogen III is generated. In mitochondria CPG is then transformed into protoporphyrinogen, which is then further oxidized to protoporphyrins. Finally, iron is incorporated to generate heme. In each specific form of porphyria (in blue) the activity of a specific enzyme (in red) in the heme biosynthetic pathway is defective and leads to accumulation of pathway intermediates. Iron (Fe2+) is involved in ALAS2 regulation via the IRE-IRP system, in FECH activity and possibly in UROD activity. Abbreviations: ALA, 5-aminolevulinic acid; ALAS, 5-aminolevulinic acid synthase; ALA-D, 5-aminolevulinic acid dehydratase; PBG, porphobilinogen; HMB, hydroxymethylbilane; PBG-D, porphobilinogen deaminase; UroPIII, uroporphyrinogen III; UROS, uroporphyrinogen III synthase; CPPIII, coproporphyrinogen III; UROD, uroporphyrinogen III decarboxylase (UROD); CPOX, coproporphyrinogen oxidase ProtoIX, protoporphyrinogen IX, PPOX, protoporphyrinogen oxidase; PPIX, protoporphyrin IX (PPIX); FECH, ferrochelatase. XLPP, X-linked protoporphyria; ALADDP, 5-aminolevulinic acid dehydratase deficiency porphyria; AIP, acute intermittent porphyria; CEP, congenital erythropoietic porphyria; PCT, porphyria cutanea tarda; HEP, hepato-erythropoietic porphyria; HCP, hereditary coproporphyria; VP, variegate porphyria; EPP, erythropoietic protoporphyria. IRP, iron regulatory protein; Ox, oxidative stress.

It is now well recognized that iron can influence heme metabolism at different levels (Figure 2).

Firstly, ALAS2 is regulated by iron at the post-transcriptional level since its mRNA contains a 5′-IRE in its untranslated region; when there is a scarcity of intracellular iron, IRPs binds to the 5′-IRE, blocking ALAS2 translation. Secondly, FECH is actually an iron–sulfur-cluster protein, which is therefore dependent both on the availability of iron and the machinery involved in iron–sulfur-cluster biogenesis: it has in fact been shown that a reduction of its levels occurs in experimental models of iron-deficient erythropoiesis (i.e., in IRP −/− mice) and in conditions of impaired iron–sulfur-cluster synthesis/regulation (i.e., in myocytes of patients with ISCU myopathy) [41].

FECH has also been shown to form complexes with Mfrn and Abcb10 in mouse models of erythroleukemia, with the possible effect of directing iron to enhance heme synthesis [42].

Moreover, in macrophages, the overall ratio of iron released by the cell is regulated not only by systemic signals through the hormone hepcidin, but also in a cell-autonomously regulated response to erythrophagocytosis, in which Fpn expression is increased by both heme and iron derived from destroyed erythrocytes [43].

It is therefore clear how heme biosynthesis depends on iron, since the latter is not only required for incorporation into the PPIX ring but also controls the synthesis and activity of ALAS2 and FECH.

## 4. Iron in Erythropoietic Porphyria

Erythropoietic protoporphyria (EPP) is a rare autosomal recessive disorder caused by the moderate to severe deficiency (<20% activity) of ferrochelatase (FECH). Homozygous or compound heterozygous mutations of the FECH gene have in fact been identified in a small number of patients [44]. In another 5–10% of cases, the defect is a gain-of-function mutation in the aminolevulinate synthase 2 (ALAS2) gene, which is encoded on the X chromosome and therefore determines the X-linked protoporphyria (XLEPP).

EPP is characterized by severe photo-toxicity induced by accumulation of PPIX in the skin, with development of redness, swelling and lesions in the affected areas and a systemic inflammatory response if the person remains exposed to the noxious agent (sunlight or strong light). This greatly impacts the quality of life of patients, who have been forced to avoid sunlight since infancy. Other potential major complications of EPP/XLP are the development of symptomatic gallstones and/or acute or chronic liver disease (i.e., protoporphyric hepatopathy) due to the accumulation of protoporphyrins in the liver. As for blood-test alterations, EPP patients frequently present with a microcytic, hypochromic anemia, which is consistent with a pattern of iron-deficiency [45,46] or impaired hemoglobin production.

There has long been debate on the role of iron supplementation in such patients. In fact, since iron is required for FECH activity and heme completion, it would be reasonable to suppose that iron supplementation would facilitate the deficient FECH activity, reduce the amount of accumulated PPIX and therefore ameliorate the phenotype in such patients. Indeed, in some case reports, iron supplementation has been shown to reduce PPIX levels in the erythrocytes and plasma of subjects with EPP [47,48]. However, its association with the exacerbation and worsening of symptoms has also been reported [49,50], likely due to the presence of a 5′-IRE in ALAS2 mRNA, which would be bound by IRP under conditions of iron scarcity or released, with consequently increased AlLAS2 levels and activity, under conditions of iron availability [51].

Furthermore, Barman-Aksozen et al. described three EPP patients (2 females and 1 male with FECH-related EPP) with decreased PPIX levels in red blood cells when they were in an iron-deficient status, with worsening microcytic, hypochromic anemia. They also documented a significant increase in ALAS2 mRNA and protein amount. To test the hypothesis of a connection between FECH deficiency and ALAS2 over-expression, FECH was inhibited in cultured cells and a subsequent increase in ALAS2 mRNA was shown, leading to the plausible conclusion that a deficiency in FECH leads to a secondary increase in ALAS2 expression [51]. Overall, these data do not support the indiscriminate regular iron administration to all patients with EPP but rather a tailored iron supplementation for iron-deficient patients, especially when anemic, with a biochemical and clinical surveillance for potential detrimental effects related to biosynthetic-intermediate accumulation.

Additionally, it can be hypothesized that the inflammatory state following the episodic cutaneous reactions could trigger hepcidin expression, leading to reduced circulatory-iron levels.

In a small case series of nine patients with EPP and one patient with XLP, Bossi et al. demonstrated a higher prevalence of iron deficiency with or without anemia (5/10 had low Ft levels (<25 ng/mL), 8/10 had low Tf saturation; 2/10 had low Hb and hematocrit per sex and age). The only female EPP subject proved to be iron deficient (serum ferritin = 4 ng/mL; transferrin saturation = 5%). The EPP patient with higher circulating iron also had classical hereditary hemochromatosis (HFE C282Y+/+). Serum and urine hepcidin levels were lower in subjects with EPP/XLP compared to healthy volunteers, and no clear relationship with serum ferritin was observed. More importantly, it was shown that intestinal adsorption of ferrous sulfate was not altered in EPP and XLP patients [52], excluding a strong inhibition of iron release form enterocytes by hepcidin and suggesting that other still-unknown factors must be in play to account for the iron-deficient phenotype. Furthermore, these preliminary data indicate that hepcidin levels are mainly driven by anemia and iron deficiency in EPP patients, which is in line with the concept that these two pathological conditions are hierarchically prevalent over inflammatory signals in hepcidin regulation in humans [53].

The hypothesis of ACD due to EPP was instead supported by an EPP mouse model in which the affected animals had an identical total-body-iron content to their control littermates but showed abnormal iron distribution in their bodies [54]. In FECH-deficient mice, most of the iron was stored in the enlarged spleen, whereas in the liver, kidney, and heart, iron content was decreased. Hepcidin expression in the liver of FECH-deficient animals was not suppressed, although these animals exhibited severe anemia, suggesting that erythropoiesis was not restricted by an absolute iron deficiency but likely limited by iron compartmentalization (i.e., functional iron deficiency), and/or the presence of strong up-regulators of hepcidin in this experimental setting. In addition, those animals showed a markedly increased transferrin expression (a typical feature of iron-deficient anemia, whereas inflammation down-regulates transferrin expression) and concomitantly decreased transferrin saturation (which is associated with both iron-deficient and inflammatory anemia), with overall normal serum iron.

In a French cohort of 55 EPP patients, Delaby et al. noted a mild hypochromic microcytic anemia and thrombocytopenia, but normal serum iron and soluble transferrin receptor (sTfR). A positive correlation between erythrocyte PPIX and Tf levels was reported, suggesting a positive effect of PPIX on the synthesis on Tf, which could facilitate the mobilization of tissue-iron stores to meet the erythropoiesis requirement and therefore a possible role of the PPIX–transferrin pathway in the regulation of iron distribution between organs [45]. This hypothesis might also explain the observation seen in the above murine model.

In another study, 178 patients with dominant EPP were evaluated; erythropoiesis was impaired in all patients, with 48% of women and 33% of men satisfying the criteria for mild microcytic, hypochromic anemia. Iron stores in the form of serum ferritin were decreased compared to the controls, with normal levels of serum iron and sTfR1, suggesting that erythropoiesis was not limited by iron supply [55].

A recent investigation of 67 EPP patients (51 Italian: 35 male and 16 female; 16 Swiss: 5 male and 11 female) compared to 21 healthy volunteers excluded the presence of anemia of chronic disease (ACD) in such patients, who had significantly decreased levels of hepcidin compared to the healthy volunteers, supporting the hypothesis of an absolute iron deficiency, possibly secondary to an inefficient iron adsorption at the intestinal level (through a hepcidin-independent mechanism) or to an undetected iron loss [56].

Overall, these data suggest that other, hepcidin-independent mechanisms could be at least partially responsible for the iron deficiency in such diseases. Furthermore, absolute and functional iron deficiency could be alternatively or concomitantly present in EPP patients, and the response to iron supplementation could be influenced by their relative weight and by the patient’s genetics (including variants in FECH, ALAS2 and other genes involved in iron homeostasis and heme metabolism).

## 5. Iron in Congenital Erythropoietic Porphyria

Congenital erythropoietic porphyria (CEP) is an autosomal recessive disease caused by mutations in the uroporphyrinogen III synthase (UROS) gene, with the consequent nonenzymatic conversion of hydroxymethylbilane to isomer I porphyrin metabolites, which accumulate in red blood cells and their late precursors, resulting in ineffective erythropoiesis, hemolysis and splenomegaly and, when disseminated into the tissues and skin, severe photosensitivity.

More than 45 mutations of UROS have been founds with other factors that can influence disease phenotype; particularly, combined mutations of ALAS2 have been associated with more severe CEP symptoms [57].

Beyond sunlight avoidance, the suppression of bone-marrow activity using different strategies was attempted in the past, such as multiple transfusions and the use of hydroxyurea; bone marrow transplants have resulted in the successful reduction of symptoms in a series of patients [58,59,60]; iron chelation has been used to ameliorate the accompanying iron overload that often develops in CEP patients (both for transfusion regimen and secondary to hemolytic anemia).

Additionally, considering the iron-dependent post-transcriptional regulation of ALAS2, the hypothesis that iron chelation with deferiprone could decrease ALAS2 expression via IRE/IRP was tested both in vitro and in a murine model of CEP, proving its efficiency at reducing porphyrin production; porphyrin accumulation progressively decreased in red blood cells and urine, and skin photosensitivity ameliorated in CEP mice treated with deferiprone (1 or 3 mg/mL in drinking water) for 26 weeks. Hemolysis and iron overload improved upon iron chelation as well, with a full correction of anemia in the CEP mice treated at the highest dose of deferiprone [60] indicating that a main effect of iron chelation is ALAS2 down-regulation. Another explanation might be related to the removal of the inhibitory effects on erythropoiesis [61].

## 6. Iron in Porphyria Cutanea Tarda

Porphyria cutanea tarda (PCT) is the most common type of porphyria worldwide, with an incidence between 20,000–70,000, and it encompasses a group of disorders caused by an insufficient/altered UROD enzymatic activity. Such deficiency leads to an accumulation of porphyrins (URO and 7-carboxyl porphyrins) in the liver.

The most frequent PCT is the acquired/sporadic type, which accounts for 75–80% of cases, in which the deficiency of UROD is limited to hepatocytes; affected patients usually present with other known risk or triggering factors such as other genetic factors (i.e., HFE mutations), viral infections (i.e., HCV-hepatitis) and exposure to certain chemical substances (i.e., alcohol, smoking, estrogens) [62].

A second type of PCT, which accounts for 20–30% of cases, is caused by mutations of the uroporphyrinogen III decarboxylase (UROD) gene in all tissues; the gene defect is transmitted in an autosomal-dominant manner with incomplete penetrance [63]. There is also a third type, which is very rare and is characterized by an apparently genetic predisposition that leads to decreased UROD activity in hepatocytes [62].

The pathogenesis of PCT is complex, but hereditary or acquired factors that lead to increased oxidative stress and hepatic-iron loading are critical in producing the clinical expression of the first and second forms of the disease [63].

From the clinical point of view, cutaneous lesions on the sunlight-exposed areas, particularly the hands and face, are the only consistent clinical feature of PCT. The most common lesions are superficial erosions from increased mechanical fragility of the skin, subepidermal bullae, hypertrichosis, and pigmentation. The age at presentation is usually lower for the familial type, while the sporadic type presents more frequently at middle age, with variations in gender likely reflecting differences in exposure to provoking factors, particularly alcohol or estrogenic therapy [64].

Liver histopathology includes the red fluorescence of unfixed hepatic tissue (as in various types of porphyria), necrosis, inflammation, and varying degrees of siderosis or steatosis.

At least 80% of patients with PCT show some degree of hepatic siderosis, especially of periportal hepatocytes [65], and total-body-iron stores are increased in 60–65% of patients [66].

Therefore, iron seems to play a role in the development of type I PCT; an imbalance in iron homeostasis may provide an oxidative environment in hepatocytes, contributing to the generation of a UROD inhibitor, likely uroporphomethene, which causes the expression of uroporphyria in mice and PCT in humans [67]. Despite the importance of liver siderosis in PCT, iron by itself is insufficient to cause uroporphyrin overproduction in the absence of other predisposing factors, emphasizing the multifactorial pathogenesis of PCT [63].

Therefore, conditions linked to increased iron accumulation, especially in the liver, have been studied and seen as possible co-risk factors for PCT development.

The prevalence of *HFE* mutations in PCT subjects has been evaluated with contrasting results in different geographical regions, although the net prevalence of both p.Cys282TyrY and p.His63Asp variants seem to be higher in affected patients compared to the general population [68,69]. Animal models with a disrupted hemochromatosis gene (*Hfe −/−)* and a disruption of one of the *Urod* alleles developed uroporphyria, which is the equivalent of human PCT in mice, whereas *Urod* −/+ mice were not affected [70,71], reinforcing the concept of an iron-driven second hit.

Hepatic hepcidin may also be reduced in PCT patients without HFE mutations, suggesting that other susceptibility factors may lower the expression of this hormone and cause hepatic siderosis in PCT [72]. Additionally, other genes important for iron regulation could be involved, as indagated in a population of 74 PCT South African patients, who underwent sequencing analysis of the promoter region of four genes that are involved in iron metabolism (ceruloplasmin (*CP*), cytochrome b reductase 1 (*CYBRD1*), hepcidin antimicrobial peptide (*HAMP*) and ferroportin or solute carrier family 40 member A1 (*SLC40A1*)). Some polymorphic loci were exhibited by PCT patients, which could contribute to the disease development by affecting iron metabolism. [73] In accordance, we recently reported an enrichment of non-HFE gene variants in a population of NAFLD patients with hepatic-iron deposition; particularly, CP variants were associated with hyperferritinemia, hepatic-iron staining and fibrosis worsening [74], indicating that genes other than HFE may affect the expression and course of chronic liver disease.

Chronic hepatitis C (CHC) is another known risk factor for PCT, and it is still not clear whether this association is secondary to iron overload due to the oxidative stress that often accompanies CHC [75] or to the hepatitis C infection (HCV) infection per se, or if both factors are relevant. As matter of fact, HCV-induced reactive oxygen species have been shown to down-regulate hepcidin transcription through inhibition of C/EBPalpha DNA binding activity in murine models, leading to increased duodenal iron transport and macrophage iron release, causing hepatic-iron accumulation [76]. In agreement, HCV infection appeared to down-regulate hepcidin expression in patients who developed PCT [72].

A similar consideration can be made for alcohol, which is a common susceptibility factor for PCT development [77]. Alcohol is a hepatotoxin that generates toxic metabolites able to induce liver damage via different mechanisms such as oxidative stress, endotoxin production, impaired immunity, hypoxia, and endoplasmic-reticulum malfunction [78]. Interestingly, alcohol has been associated with hepcidin down-regulation via oxidative stress both in hepatoma cell lines and rodents [79], indicating another way for alcohol to trigger or enhance the clinical expression of PCT. Alcohol may also contribute to iron-metabolism derangement via other mechanisms, including acute and chronic liver injury, the increased ability of desialylated transferrin to deliver iron to the liver, ineffective erythropoiesis and chronic hemolysis.

Overall, the data indicate that genetic and acquired factors capable of inducing iron overload in the liver, including through down-regulation of hepcidin expression, contribute to the overt clinical expression of PCT. Consistent with this, iron depletion (with phlebotomy as the first-line treatment) or the treatment of underlying disease as well (antiviral therapy in case of HCV infection, alcohol withdrawn), have been associated with the gradual improvement and clinical remission of disease expression in patients diagnosed with PCT [63].

## 7. Iron in Acute Hepatic Porphyrias

The acute hepatic porphyrias (AHPs) include four of the rarest types of inherited porphyrias: acute intermittent porphyria (AIP), variegate porphyria, hereditary coproporphyria, and porphyria due to severe deficiency of ALA dehydratase (ADP).

Of these, AIP is by far the most prevalent. It is due to mutations of the HMBS gene (transmitted in an autosomal-dominant manner with low penetrance), which causes a deficient activity of porphobilinogen deaminase (PBG-D; the third enzyme in the heme biosynthesis pathway), with consequent accumulation of the precursors of ALA and PBG in the liver [80].

AIP is clinically characterized by some of the most serious manifestations of porphyrias, like recurring neurovisceral acute porphyric attacks (APAs), which can be triggered by certain drugs, starvation, inflammation, stress and hormones, and can be lethal if untreated.

As HMBS is less abundant than the other enzymes of the heme synthetic pathway, it becomes rate-limiting when ALAS1 is markedly increased. As a result, the porphyrin precursors are overproduced, and they markedly accumulate. The liver is the primary target of damage and, accordingly, affected patients tend to have altered-liver-function tests and a higher incidence of hepatocellular carcinoma [81].

Although recently givosiran, an siRNA directed specifically at hepatic ALA synthase-1 has been approved for the prevention of recurrent acute attacks in severely affected patients with AHPs, up to now, the standard therapy for APAs has been the intravenous administration of heme-arginate, in order to create negative feedback for ALAS1 activity. Patients who experienced recurring APAs tended to receive repeated infusions of hemin as a prophylactic treatment, with the consequent increased risk of iron overload [82]. In these patients, iron overload due to heme administration can contribute to hepatic fibrosis and the development of hepatocellular carcinoma [82], as seen in other chronic liver diseases of other origins with intercurrent iron accumulation [83].

Other than heminic treatment, mechanisms activated in response to starvation and linking APA and iron metabolism could be involved. It has been demonstrated that a transcriptional coactivator, peroxisome-proliferator-activated receptor-gamma coactivator-1 (PGC-1alpha), is a key player in the induction of porphyria by fasting. In fact, in a fasted state, the hepatic expression of PGC-1alpha is induced, which then acts as a coactivator for the transcription factors nuclear respiratory factor 1 (NRF-1) and forkhead box protein O1 (FOXO1), resulting in increased ALAS-1 expression and an increased risk of an APA.

On the other hand, it has been previously shown that during starvation PGC-1alpha also binds to the hepcidin gene promoter, as well as c-AMP responsive element binding protein 3 like 3 (Creb3l3), leading to increased hepcidin expression, ferroportin degradation and consequent hypoferremia and iron retention in the liver [84].

Thus, starvation could both trigger APAs through ALAs-1 induction and favor iron-status derangement and liver-iron damage via a common molecular co-activator. Indeed, it is not surprising that molecular crossroads exist between energy homeostasis, heme-protein synthesis and iron metabolism, and that the fine regulation of shared key players is crucial.

The liver-iron-overload issue in AIP patients is not trivial or outdated as many patients have already undergone long-term hemin treatment, and probably not all patients with AIP will be suitable or tolerant of long-life siRNA therapeutic strategies in the future.

## 8. Other Evidence from Experimental Models

Spontaneous mouse models for porphyrias have not yet emerged; however, mouse models have been created for all of the main types of hereditary porphyrias, mostly via gene targeting, with the exception of XLP and ADP, although homozygous knockout mice for any of the heme biosynthetic genes are embryonically lethal [71].

Mouse models have also been used to test the hypothesis that defects in other steps of iron metabolism or transport could predispose the development of porphyrias.

For instance, it has been shown how mitoferrin activity is necessary for heme synthesis in the case of increased porphyrin production: selective hepatic deletion of Mfrn1 in mice has no phenotype or biochemical effect under normal conditions but, in the presence of increased porphyrin synthesis, leads to a decreased ability to convert PPIX into heme and, therefore, to PPIX accumulation. This is due to an insufficient iron availability within the mitochondria, which is not covered by Mfrn2 activity, leading to protoporphyria, cholestasis, and bridging cirrhosis [85]. Additionally, the accumulation of porphyrins in animal models that are deficient in Mfrn1 in the erythroid precursors seems to be prevented by IRP1; an impaired mitochondrial [Fe–S] cluster biogenesis in Mfrn1 −/− cells results in an elevated IRP1 RNA-binding that attenuates ALAS2 mRNA translation and protoporphyrin accumulation [86].

Furthermore, a mouse model of IRP2 deficiency has been described, presenting with no abnormalities in the liver, a microcytic anemia due to iron-limited erythropoiesis (with a reduced TfR2 expression in erythroid precursors and absent iron stores in the bone marrow, but with normal Tf saturation and an overexpressed circulating ferritin) and a marked overexpression of ALAS-2 resulting from the loss of its IRP2-mediated translational repression, and therefore an increased free protoporphyrin IX and zinc protoporphyrin [87].

Mouse models have so far contributed to a better understanding of porphyria pathogenesis and molecular mechanisms linking iron homeostasis to erythropoiesis. Nevertheless, since rodents do not have the exact same mechanisms involved in erythropoietic processes and systemic iron regulation of humans [37], murine models do not accurately recapitulate human porphyria pathophysiology.

Moreover, iron supplementation or depletion has not been extensively evaluated as a therapeutic approach in murine models of porphyrias, likely due to the difficulty of mirroring human physiopathology and the need for more targeted therapeutic strategies in such complex metabolic diseases.

## 9. Conclusions

Although the landscape of therapeutic strategies for porphyrias is rapidly changing and significantly expanding, patients still suffer from complications and a poor quality of life.

The most recent advances in the knowledge of heme biosynthesis and iron homeostasis have highlighted novel concepts and future research areas in order to better understand the pathogenesis, clinical manifestations and complications of porphyrias. Furthermore, the molecular and biochemical intersections between nutrient and energetic metabolism, iron homeostasis and heme biosynthesis are increasingly recognized. Thus, a system-biology approach based on these molecular and metabolic links is preferable in order to understand when, how and where iron can be ‘friend or foe’ in different forms of porphyria, in different patients or in different stages of the disease.

Due to the limitations of non-human models of the disease, patient-based phenotyping and genotyping are still needed to improve our understanding of the complexity of porphyria manifestations and natural history.

A reliable description of the level and distribution of body iron in porphyrias has not yet been provided. Additionally, the impact of iron-status modulations in the disease expression is not yet fully understood for most forms of the disease. Moreover, genetic cofactors or modifiers (including variants in iron genes) possibly affecting disease development and progression are almost entirely unexplored.

In addition to the classic biochemical parameters, more recent and accurate tools are becoming widely available to deepen the characterization of iron status and metabolism, including magnetic resonance imaging (MRI) for the detection and quantification of iron in different tissues (such as liver, spleen, heart, kidney, brain), non-standard or experimental serum measurements (such as soluble transferrin receptor, reticulocyte hemoglobin content, percentage of hypochromic red blood cells, and hepcidin), and next-generation sequencing technologies (NGS) that enable fast, cost-effective and reliable parallel analyses of multiple genes and patients.

For all these reasons, serum-iron parameters (i.e., iron, transferrin, transferrin saturation, and ferritin) should be included in the tests that are routinely performed to study and stratify such patients in clinical practice, in order to pave the way for a future use of iron-status markers as additional diagnostic tools and therapeutic targets, particularly for certain types of porphyria.

Furthermore, a systemic characterization of iron status could be performed in the reference centers for research purposes, integrating clinical information, hematological and biochemical parameters, MRI data, and genomic information, with the aim of a better understanding of the disease pathophysiology and a more tailored and effective patient management.

## Data Availability

Not applicable.
